# HLA-G 3’UTR Polymorphisms Are Linked to Susceptibility and Survival in Spanish Gastric Adenocarcinoma Patients

**DOI:** 10.3389/fimmu.2021.698438

**Published:** 2021-09-07

**Authors:** Christian Vaquero-Yuste, Ignacio Juarez, Marta Molina-Alejandre, Elisa María Molanes-López, Adrián López-Nares, Fabio Suárez-Trujillo, Alberto Gutiérrez-Calvo, Adela López-García, Inmaculada Lasa, Remedios Gómez, Eduardo Fernández-Cruz, Carmen Rodrígez-Sainz, Antonio Arnaiz-Villena, José Manuel Martín-Villa

**Affiliations:** ^1^Departamento de Inmunología, Oftalmología y ORL, Facultad de Medicina, Universidad Complutense de Madrid, Madrid, Spain; ^2^Departamento de Estadística e Investigación Operativa, Facultad de Medicina, Universidad Complutense de Madrid, Madrid, Spain; ^3^Servicio de Cirugía General y Aparato Digestivo, Hospital Universitario Príncipe de Asturias, Madrid, Spain; ^4^Instituto de Investigación Sanitaria Gregorio Marañón, Madrid, Spain

**Keywords:** HLA-G, cancer, gastric cancer, immunotherapy, immunoediting, +3142 C/G, 14bp INS/DEL, adenocarcinoma

## Abstract

HLA-G is a non-classical class I HLA molecule that induces tolerance by acting on receptors of both innate and adaptive immune cells. When overexpressed in tumors, limits surveillance by the immune system. The *HLA-G* gene shows several polymorphisms involved in mRNA and protein levels. We decided to study the implication of two polymorphisms (rs371194629; 14bp INS/DEL and rs1063320; +3142 C/G) in paired tissue samples (tumoral and non-tumoral) from 107 Spanish patients with gastric adenocarcinoma and 58 healthy control individuals, to assess the possible association of the *HLA-G* gene with gastric adenocarcinoma susceptibility, disease progression and survival. The presence of somatic mutations involving these polymorphisms was also analyzed. The frequency of the 14bp DEL allele was increased in patients (70.0%) compared to controls (57.0%, p=0.025). In addition, the haplotype formed by the combination of the 14bp DEL/+3142 C variants is also increased in patients (54.1% *vs* 44.4%, p=0.034, OR=1.74 CI95% 1.05-2.89). Kaplan-Meier analysis revealed that 14bp DEL/DEL patients showed lower 5-year life-expectancy than INS/DEL or INS/INS (p=0.041). Adjusting for TNM staging (Cox regression analysis) disclosed a significant difference in death risk (p=0.03) with an expected hazard 2.6 times higher. Finally, no somatic mutations were found when comparing these polymorphisms in tumoral *vs* non-tumoral tissues, which indicates that this is a preexisting condition in patients and not a *de novo*, tumor-restricted, event. In conclusion, the variants predominant in patients were those increasing HLA-G mRNA stability and HLA-G expression, clearly involving this molecule in gastric adenocarcinoma susceptibility, disease progression and survival and making it a potential target for immunotherapeutic approaches.

## Introduction

Gastric epithelial adenocarcinomas are the most common form of stomach tumors, representing 90% of the cases. Adenocarcinomas are usually located in the cardia (31.0%), antrum (26.0%) and body (14.0%), and their development is associated to the infection by *Helicobacter pylori*, as 84.0% of the patients have been infected with this bacterium (1). In 2020, this tumor was the 6th most frequent cancer in the world (11.1 new cases per 100.000 persons per year), and the 5th with highest mortality rate (7.7 patients per 100.000 persons per year), which made it one of the most aggressive tumors. The 5-year survival rate for this type of cancer was 29.5%, a situation that has been maintained for the last 30 years (2, 3).

Tumors with high frequency of somatic mutations, such as stomach malignancies (4), express tumor neo-antigens on their surface and are, thus, potential targets of the immune response. However, even in an immunocompetent organism, neoplastic cells develop mechanisms, such as the expression of immunomodulatory molecules as HLA-G, to evade the action of the immune system. Published works describe that HLA-G possesses a key role in the cancer immunoediting mechanism, attenuating the elimination of tumor cells (5–8).

HLA-G is a non-classical class I HLA molecule composed of a heavy chain bound to β2 microglobulin. The *HLA-G* gene, located on chromosome 6, exhibits 7 introns and 8 exons that codifies for the heavy chain. Exon 1 codes for the signal peptide, exons 2, 3 and 4 the extracellular domains α1, α2 and α3, respectively, and exons 5 and 6 the transmembrane region and the cytoplasmic domain, respectively (9). Exon 7 is transcribed in the pre-mRNA molecule, but not present in the mature-mRNA, whereas exon 8 is not translated., but in the latter lays the 3’UTR region, involved in the transcriptional regulation of the gene (10, 11).

HLA-G shows tolerogenic functions by inhibiting immunocompetent cells, through the interaction of HLA-G (whether membrane or soluble isoforms) with cognate receptors. These receptors are the immunoglobulin-like transcription receptor type 2 (ILT2, CD85j), present on NK, B, T cells and antigen-presenting cells (APCs) (12, 13); the ILT4 (CD85d) receptor (that along with ILT2, displays ITIM motifs), unique to APCs (12, 14); the killer immunoglobulin-like receptor (KIR) 2DL4, present on NK cells (15); and, finally, CD8 (16). Because of this tolerogenic role, HLA-G is involved in a wide variety of processes, such as maternal-fetal tolerance (17), organ transplantation (17), viral infections (18, 19), autoimmunity (18, 20), and cancer progression (5).

The 3’UTR region of the *HLA-G* gene shows numerous variations that can have an impact on the mRNA and, therefore, on the protein levels (10). Of these variants, rs371194629 and rs1063320 have been studied in several types of cancer and pathologies (21–23).

The rs371194629 polymorphism (14bp INS/DEL) is caused by the deletion (DEL) from the ancestral variant (INS) (10, 24) of a 14bp segment (5′-ATTTGTTCATGCCT-3’) located at the +2960 position of the 3’UTR region. This 14bp segment has been associated with both, the splicing and the stability of the mRNA (10, 25, 26), as it contains an AUUUG domain putatively exerting an AU-pentamer-like effect, decreasing mRNA stability (27). Therefore, the DEL allele provides a higher stability of the mRNA (25), associated with a high expression of HLA-G (26).

The rs1063320 polymorphism (+3142C/G), consists of the transversion of a cytosine (C, the ancestral variant) to guanine (G) at position +3142 of the 3’UTR region, modulating the affinity of 148a, 148b and 152 miRNAs (known to either favor the direct mRNA degradation or block mRNA to protein translation) for this region (28–30). Should a C be found at position +3142, miRNAs affinity will decrease, increasing the mRNA availability and the production of HLA-G (10, 11).

Other authors have described the expression of HLA-G in gastric tumors (31), therefore, studying the polymorphisms related to the expression levels (such as 14bp INS/DEL and +3142 C/G) of this molecule could serve to identify new genetic markers involved in the risk and evolution of this pathology.

Therefore, we decided to study the influence of the aforementioned polymorphisms and the combined haplotypes thereof in paired tissue samples (tumoral and non-tumoral) from a group of patients with gastric adenocarcinoma, and compare the frequencies achieved with that of a control population. This approach will allow us to determine whether the HLA-G variants may be adequate gastric adenocarcinoma risk markers and whether somatic mutations take place in tumoral, but not healthy, gastric tissue.

## Patients, Materials, and Methods

Samples used in this study were obtained from the Servicio de Cirugía General y Digestiva, Hospital Príncipe de Asturias (Alcalá de Henares - Madrid) and sent to the Departamento de Inmunología (Facultad de Medicina. Universidad Complutense de Madrid - Madrid), where they were processed.

### Patients

One hundred and seven Spanish patients diagnosed with gastric adenocarcinoma were included in this study. Patients were classified according to the TNM staging criteria (stages I through IV) (32) (**Table 1**).

**Table 1:** Demographic and clinical characteristics of patients.

Total patients (N = 107)				
**Sex**				**Median age**
**Male**	**Female**	**No data**		70 (33 - 89)
62 (58.0%)	41 (38.3%)	4 (3.7%)		
**Stage**				
**I**	**II**	**III**	**IV**	**No data**
24 (22.4%)	36 (33.7%)	21 (19.6%)	22 (20.6%)	4 (3.7%)
**Tumor location**				
**Cardia**	**Body**	**Fundus**	**Antrum**	**No data**
11 (10.3%)	34 (31.8%)	15 (14.0%)	41 (38.3%)	6 (5.6%)
**Tumor type**				
**Diffuse**	**Intestinal**	**Undifferentiated**		**No data**
34 (31.8%)	52 (48.6%)	15 (14.0%)		6 (5.6%)

The inclusion criteria for this study were patients (women or men) over 18 years old with gastric adenocarcinoma, stratified according to UICC/AJCC criteria (7th edition 2009) (32), in any TNM stage and Karnofsky index >70% or performance status ≤ 2. All patients signed an informed consent before their inclusion in the study, and all samples were anonymized upon arrival to the laboratory.

The exclusion criteria included resecability of the primary tumor, coexistence with other neoplastic diseases, pregnancy and severe alterations of hepatic, cardiovascular o renal function.

Comorbidities included in this study were ULCUS (21%), hypertension (33%), cardiopathy (8%) pulmonary disease (21%), liver disease (2%), renal disease (2%), cerebrovascular disease (4%), diabetes (17%), smoker (19%) and alcoholism (4%).

### Tissue Samples

Tumoral (T) and distal, non-tumoral (NT), gastric tissue samples were obtained from each patient upon surgery. A total of 214 tissue samples (T+NT) were available for the study.

### Controls

A total of 58 sex-and age-matched Spanish healthy donors, from the same geographic region as the patients, were included as controls. DNA was obtained from blood or saliva samples, as described in the following section.

### Genomic DNA Extraction

DNA isolation, both from blood and tissue, was carried out by using the Illustra Nucleon BACC (GE Healthcare) kit, following the manufacturer’s instructions. In the case of tissue specimens, fragments of 25 mg were mechanically disrupted, subjected to proteinase K treatment, and followed by the DNA precipitation protocol included in the kit. DNA from saliva control samples were extracted using the Oragene DNA 500 kit (DNA Genotek) and purified with PrepIT-L2P (DNA Genotek).

The concentration and quality of DNA extracted per sample was determined by spectrophotometric methods in a NanoDropOne (ThermoScientific).

### Analysis of the 14bp Polymorphism

**Figure 1:**
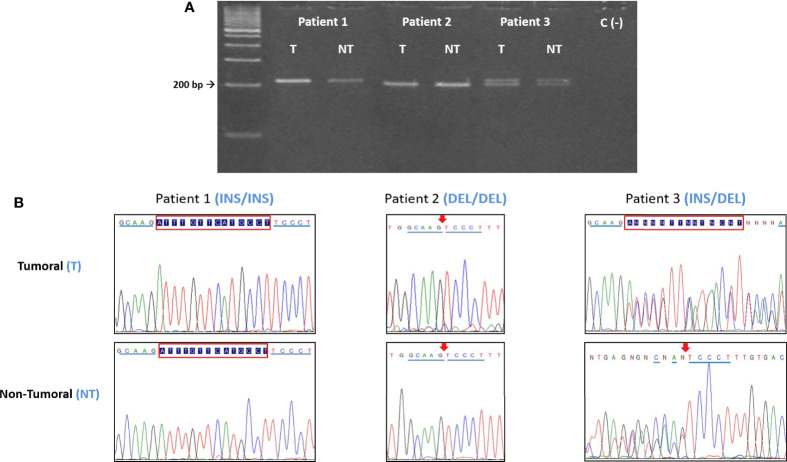
**(A)** Electrophoretic and **(B)** sequencing analysis of the 14bp INS/DEL polymorphism. For each patient, the tumoral (T) DNA sample was analyzed paired to its non-tumoral (NT) counterpart. In this example, Patient 1 is INS homozygous, Patient 2 is DEL homozygous and Patient 3 is heterozygous (INS/DEL). In all the instances the T and NT samples from each patient have the same polymorphic variant, indicating that there are no somatic mutations. INS = 224bp, DEL = 210bp. Red box= 14bp insertion. Arrow = 14bp deletion.

The region of exon 8 containing the 14bp polymorphism was amplified by PCR, using primers and conditions previously published (33, 34) (**Table 2A**), further confirmed using the NCBI Blast tool. Amplified products (224bp, INS variant or 210bp, DEL variant) were resolved by electrophoretic analysis in 3% agarose gels for 80 min at 90V (**Figure 1A**).

**Table 2:** Primers and conditions used **(A)** for the 14bp INS/DEL polymorphism and **(B)** for the +3142 C/G polymorphism (33–35).

(A)	Polymorphism	Primers		PCR conditions				
	**14bp INS/DEL**(*30 cycles)	**Forward**	**Reverse**	**Denaturalization**		**Annealing**	**Elongation**	
		5’-**GTGATGGG CTGTTTAAA GTGTCACC** -3’	5’- **GGAAGGA ATGCAGTTC AGCATGA** -3’	94°C		64°C	72°C	
				2 min	30 sec	60 sec	60 sec	10 min
**(B)**	**Polymorphism**	**Primers**		**PCR conditions**				
	**+3142 C/G** (*32 cycles)	**Forward**	**Reverse**	**Denaturalization**		**Annealing**	**Elongation**	
		5’- **CATGCTG AACTGCAT TCCTTC**C -3’	5’- **CTGGTGG GACAAGGT TCTACTG** -3’	94°C		65,5°C	72°C	
				5 min	30 sec	30 sec	60 sec	5 min

The BseSI restriction enzyme was used to analyze the latter polymorphism.

Bold values mean statistically significant values.

### Analysis of the +3142 C/G Polymorphism

**Figure 2:**
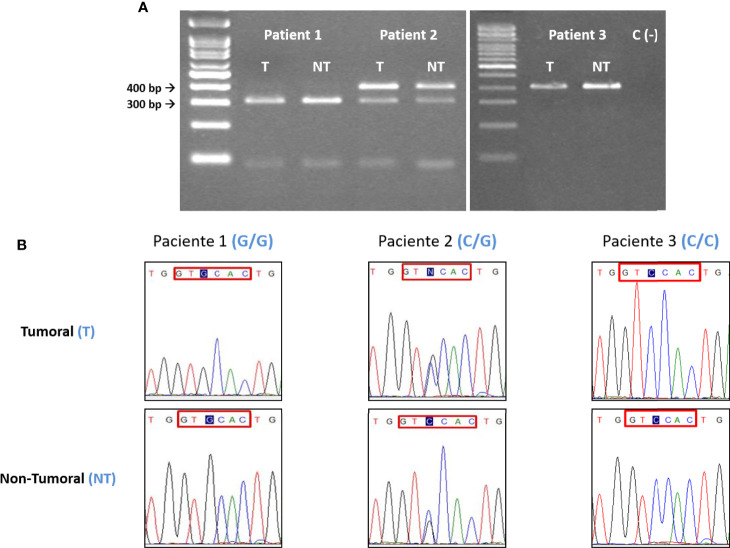
**(A)** Electrophoretic and **(B)** sequencing analysis of the +3142 C/G polymorphism. For each patient, the tumoral (T) DNA sample was analyzed paired to its non-tumoral (NT) counterpart. In this example, Patient 1 is G homozygous, Patient 2 is heterozygous (C/G) and Patient 3 is C homozygous. In all the instances the T and NT samples from each patient have the same polymorphic variant, indicating that there are no somatic mutations. C = 406bp, G = 316 and 90bp. Blue tint = +3142 SNP. Red box= BseSI restriction target. When a G is placed in the +3142 position the restriction occurs.

PCR primers and conditions used have been already published (34, 35) (**Table 2B**). The PCR product was subjected to BseSI digestion (PCR-RFLP) and resolved in a 2% agarose gel for 50 min at 90V. Effective digestion will disclose a “G” at position +3142, yielding two bands of 316bp and 90bp, whereas an undigested amplicon will indicate a “C” at this position and a single 406bp band (**Figure 2A**).

### Sequencing

DNA sequencing (Sanger method) was carried out in 272 samples (107 patient paired samples plus 58 controls), to confirm the PCR and PCR-RFLP results (**Figures 1B**, **2B**).

### Statistics

The data of the polymorphisms studied by PCR or PCR-RFLP were analyzed with the software SNPStats. This software allows to assess Hardy-Weinberg Equilibrium (exact test), chi-square test, OR estimation analysis of association between polymorphisms and disease applying logistic regression models, that consider the dominant, recessive, codominant and log-additive models of inheritance. SNPStats also allows the analysis of linkage disequilibrium, using the D statistic and a correlation coefficient, and the analysis of haplotypes (EM algorithm) (36, 37).

Kaplan-Meier method was used to estimate the 5-year survival function of patients with gastric cancer and different genetic factors (GraphPad Prism 8.0 software). Multivariate Cox regression models were used to simultaneously assess the effect of genetic factors and other factors such as comorbidities, clinical features and demographic characteristics on 5-year survival of patients (software R). For all the Cox regression fits, the individual and global Schoenfeld test indicated that no covariate in the model nor the model as a whole violate the Proportional Hazard assumption, meaning that the hazard ratio stays constant over time (38).

P-values below 0.05 were considered statistically significant. As two polymorphisms were considered, the Holm-Bonferroni (HB) sequential correction method for multiple testing was applied to the statistical analyses when required. The HB method compares the k-ranked p-value to the nominal significance level (0.05) divided by (n-k+1), where in this case n = 2 (the number of polymorphisms) and k = 1 and 2.

## Results

The clinical characteristics of patients are shown in **Table 1**.

### Genetic Analysis

Hardy-Weinberg equilibrium was confirmed for both polymorphisms and groups of individuals included in the study (data not shown).

#### 14bp INS/DEL Polymorphism

Comparing the frequency of the 14bp INS/DEL polymorphism in patients and our control group yielded statistically significant differences.

The 14bp DEL variant was the most frequent in the group of patients (70.0%) compared to the control group (57.0%, p=0.025 see **Table 3A**). Genotype distribution (INS/INS, INS/DEL, DEL/DEL) showed no statistically significant differences between both groups (P=0.089, **Table 3B**), although DEL/DEL individuals were more abundant (50.9%) in patients than in controls (34.5%).

**Table 3:** Polymorphism 14bp INS/DEL.

(A)		Allelic frequencies (N=161)						
		**Allele**	**N Ctrl (%)**		**N patients (%)**		**P-value**	
		**INS**	47 (43.0%)		64 (30.0%)		**0.025**	
		**DEL**	63 (57.0%)		148 (70.0%)			
**(B)**		**Genotypic frequencies (N=161)**						
		**Genotype**	**N Ctrl (%)**		**N patients (%)**		**P-value**	
		**INS/INS**	11 (20.0%)		12 (11.3%)		0.089	
		**INS/DEL**	25 (45.5%)		40 (37.7%)		
		**DEL/DEL**	19 (34.5%)		55 (50.9%)		
**(C)**	**Distribution models (N=161)**							
	**Model**	**Genotype**	**Ctrl**	**Patients**	**OR (95% CI)**	**P-value**	**AIC**	**BIC**
	**Dominant**	INS/INS	11 (20.0%)	12 (11.3%)	1.00	0.14	208.6	214.8
		INS/DEL-DEL/DEL	44 (80.0%)	94 (88.7%)	1.96 (0.80-4.78)			
	**Recessive**	INS/INS-INS/DEL	36 (65.5%)	52 (49.1%)	1.00	**0.046**	206.8	212.9
		DEL/DEL	19 (34.5%)	54 (50.9%)	1.97 (1.00-3.86)			
	**Codominant**	INS/INS	11 (20%)	12 (11.3%)	1.00	0.1	208.2	217.4
		INS/DEL	25 (45.5%)	40 (37.7%)	1.47 (0.56-3.83)			
		DEL/DEL	19 (34.5%)	54 (50.9%)	2.61 (0.99-6.88)			
	Log-additive*	—	—	—	**1.65 (1.04-2.61)**	**0.034**	**206.2**	**212.4**

**(A)** Allelic frequencies. **(B)** Genotypic frequencies. **(C)** Distribution models.

*DEL as variant of interest.

Bold values mean statistically significant values.

The best fit model for this polymorphism, based on the Akaike Information Criterion (AIC) and Bayesian Information Criterion (BIC) values, was the log-additive model, where having each copy of DEL modifies the risk of developing gastric cancer in an additive form. DEL-allele bearers showed a higher risk of developing gastric cancer (p=0.034 OR 1.65, CI95% 1.04-2.61, see **Table 3C**).

As already mentioned, the DEL variant provides greater stability to the HLA-G mRNA, and, hence, yields higher levels of the protein, favoring tumor progression.

#### +3142 C/G Polymorphism

Although no significant difference was observed in the distribution of the alleles of this polymorphism (**Table 4A**), when considering the inheritance models of the different genotypes, significant differences were found. Based on the AIC and BIC values, the recessive inheritance model is the one that best fits the data obtained, with an increased frequency of C/C individuals in the group of patients (30.8%) compared to controls (14.3%, p=0.017, OR=2.68, CI95% 1.14-6.28, see **Table 4C**).

**Table 4:** Polymorphism +3142 C/G.

(A)		Allelic frequencies (N=163)						
		**Allele**	**N Ctrl (%)**		**N patients (%)**		**P-value**	
		**G**	62 (55.0%)		97 (45.0%)		0.085	
		**C**	50 (45.0%)		117 (55.0%)			
**(B)**		**Genotypic frequencies (N=163)**						
		**Genotype**	**N Ctrl (%)**		**N patients (%)**		**P-value**	
		**G/G**	14 (25.0%)		23 (21.5%)		0.067	
		**C/G**	34 (60.7%)		51 (47.7%)		
		**C/C**	8 (14.3%)		33 (30.8%)		
**(C)**	**Distribution models (N=163)**							
	**Model**	**Genotype**	**Ctrl**	**Patients**	**OR (95% CI)**	**P-value**	**AIC**	**BIC**
	**Dominant**	G/G	14 (25.0%)	23 (21.5%)	1.00	0.61	213.5	219.7
		C/G-C/C	42 (75.0%)	84 (78.5%)	1.22 (0.57-2.60)			
	**Recessive**	G/G-C/G	48 (85.7%)	74 (69.2%)	1.00	**0.017**	**208**	**214.2**
		C/C	8 (14.3%)	33 (30.8%)	**2.68 (1.14-6.28)**			
	**Codominant**	G/G	14 (25.0%)	23 (21.5%)	1.00	0.056	210	219.2
		C/G	34 (60.7%)	51 (47.7%)	0.91 (0.41-2.02)			
		C/C	8 (14.3%)	33 (30.8%)	2.51 (0.91-6.96)			
	Log-additive*	—	—	—	1.53 (0.95-2.47)	0.078	210.6	216.8

**(A)** Allelic frequencies. **(B)** Genotypic frequencies. **(C)** Distribution models.

*C as variant of interest.

Bold values mean statistically significant values.

Like the DEL variant of the previous polymorphism, the +3142 C variant would yield higher levels of the final protein, possibly favoring tumor progression.

Based on the Holm-Bonferroni sequential correction, significant associations are still detected in both +3142 (through the recessive model with the first-ranked p-value = 0.017<0.025) and 14bp (through the log-additive model with the second-ranked p-value=0.034<0.05) polymorphisms.

#### Haplotype Formed by the 14bp INS/DEL and +3142 C/G Polymorphisms

These two polymorphisms lie in close vicinity (182bp) and are in linkage disequilibrium, according to the values obtained with the SNPStats software (D’=0.98, r=0.73, **Table 5**), forming haplotypes.

**Table 5:** Haplotype frequencies estimation and distribution.

D’ statistic	0.9794			r statistic	0.728
**Haplotype frequencies estimation (N=165)**					
**14bp INS/DEL**	**+3142 C/G**	**Ctrl**	**Patients**	**OR (IC 95%)**	**P-value**
**INS**	**G**	42.5%	30.0%	1.00	—
**DEL**	**C**	**44.4%**	**54.1%**	**1.74 (1.05 – 2.89)**	**0.034**
**DEL**	**G**	13.1%	15.4%	1.53 (0.79 – 2.97)	0.71
**INS**	**C**	0.0%	0.5%	—	<0.0001

Bold values mean statistically significant values.

The frequency of the 14bp-DEL/+3142-C (DEL/C) haplotype (**Table 5**) is higher in patients (54.1%) than in control individuals (44.4%, p=0.034, OR=1.74 CI95% 1.05-2.89). These results are consistent with the allele frequencies obtained for each polymorphism, as the variants producing higher expression of HLA-G are more frequent in patients with gastric adenocarcinoma. Therefore, this supports the results obtained at the individual level for each polymorphism and denotes DEL/C as a possible risk haplotype.

#### Somatic Mutations

The distribution of the polymorphisms studied was compared in paired (T and NT) gastric tissue samples in all patients. In no instance was a difference found between the paired tissue samples analyzed, irrespective of the polymorphisms (14bp INS/DEL or +3142 C/G) considered. To further confirm these data, blood samples (EDTA) from patients were drawn, and the results obtained matched those of tissues (results not shown).

### Survival Curves

**Figure 3:**
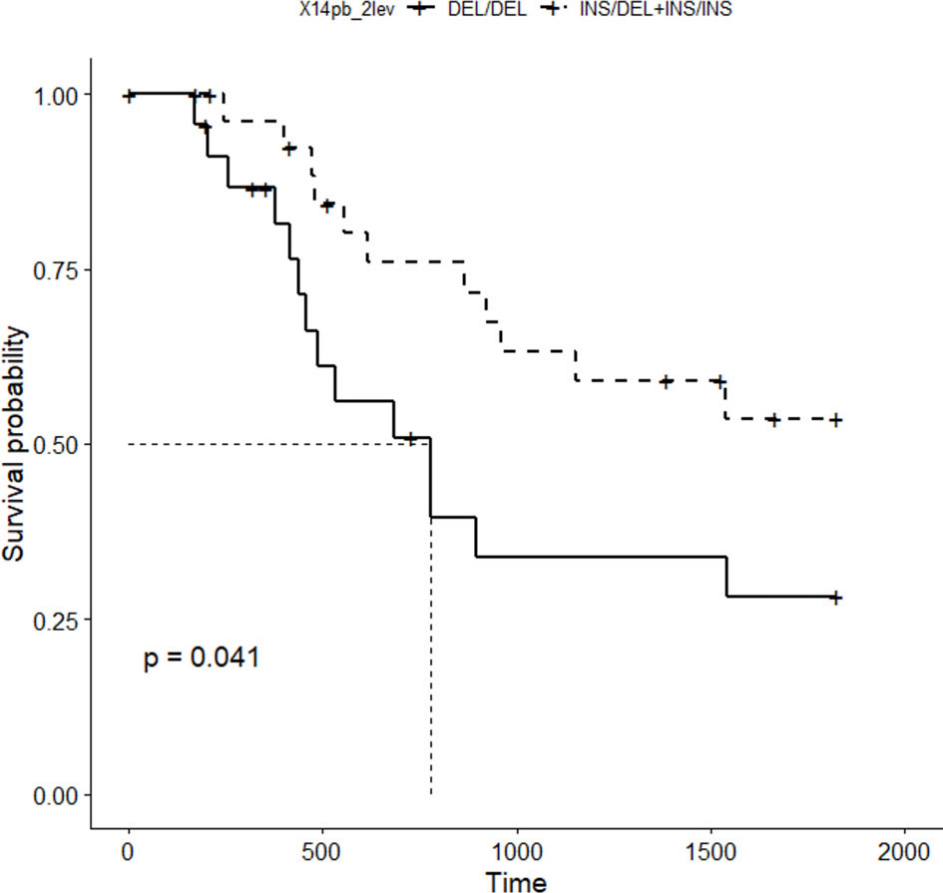
5-year disease specific survival curve (Kaplan-Meier) obtained for the 14bp polymorphism (patients were grouped in two categories: DEL/DEL, N=23 *vs* INS/DEL+INS/INS, N=29).

**Figure 4:**
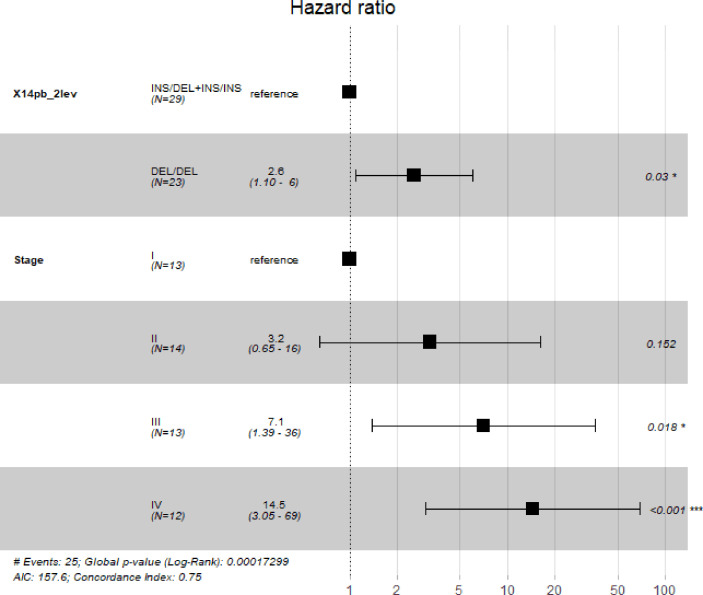
Multivariate Cox regression carried out to assess simultaneously the effect of other factors (sex, age, cancer stage, cancer location and cancer type) on 5-year survival of patients. Our analyses indicate that polymorphism 14bp is significantly associated with 5-year survival when adjusting by cancer stage. The “*”symbol means p < 0.05, and the “***” symbol means p < 0.005.

Five-year survival rate of patients with gastric cancer was calculated. Only patients enrolled in the study for at least 5 years (52 out of the 107 patients) were included. Patients were divided into two groups, according to their 14bp genotype: DEL/DEL and INS/DEL+ INS/INS, the former expressing more HLA-G than the latter, according to previous published work (39). Kaplan-Meier analysis (**Figure 3**) revealed significantly lower survival rate in patients bearing the 14bp DEL/DEL genotype (N=23) as compared to patients with either INS/DEL or INS/INS genotypes (N=29) (28% *vs* 54%, p=0.041). After adjustment for stage, multivariate Cox regression analysis (**Figure 4**) indicated a statistically significant difference in death risk (Wald test p=0.03, HR=2.6 CI95% 1.10-6.00), with an expected hazard 2.6 times higher among DEL/DEL patients as compared to patients with either INS/DEL or INS/INS genotypes. Besides, patient inclusion in stage III (Wald test p=0.018, HR 7.1 CI95% 1.39-35.56) or stage IV (Wald test p< 0.001, HR 14.5 CI95% 3.05-68.80) group was a clear statistically significant risk factor. Compared with stage I patients, the risk of death was 7.95 times higher in stage III patients and 14.48 times higher in stage IV patients.

The +3142C/G polymorphism variants yielded no significant effect on the survival of these patients (data not shown).

## Discussion

The association between HLA-G, immunoediting and cancer has been of great interest in recent years (40–42). In fact, HLA-G-mediated signaling pathway is now considered as a new therapeutic immune checkpoint, in addition to other well-established ones (41).

We thus decided to analyze the involvement of HLA-G in the development of gastric adenocarcinoma. To this end, we examined in a group of 107 patients the influence of two polymorphisms (14bp INS/DEL and +3142 C/G) that affect the stability and availability of the HLA-G mRNA and, therefore, the level of the corresponding protein. A higher frequency of the variants increasing HLA-G mRNA stability (14bp DEL and +3142 C) is expected in patients, as they will favor the development and progression of gastric adenocarcinoma. Our results clearly show that HLA-G polymorphisms are linked to gastric cancer susceptibility.

### Genetic Analysis

#### 14bp INS/DEL Polymorphism

A significant difference was found in the frequency of the DEL allele in patients compared to controls (**Table 3A**). Since already published work (9–11) showed that this variant increases HLA-G expression, we suggest that patients bearing the DEL allele will extensively display HLA-G, allowing tumor progression. These differences were also confirmed using a larger cohort of controls of Italian origin (N=245) obtained in a bibliographic search (43) (data not shown): the DEL allele was present in 57.0% of individuals, significantly different from our diseased group (p=0.001). We used an Italian population as they have a similar HLA genetic background (44). Likewise, the 14bp DEL variant was linked to the development of breast, esophageal and colorectal cancer (39, 45–47). We may conclude that the 14bp DEL variant is a potential new gastric carcinoma risk marker.

As far as inheritance models are concerned, and according to the AIC, BIC and OR values (**Table 3C**), the most appropriate model to describe the distribution of these genotypes is the log-additive, whereby only the risk variant (DEL), has an effect in disease susceptibility, which, furthermore, is additive. Thus, the presence of a single copy of DEL (as in INS/DEL individuals) implies 1.65 times the risk of suffering gastric cancer, and the presence of two copies (DEL/DEL individuals; 50.9% in patients *vs* 34.5% in controls) will further increase this risk.

Previous published research articles revealed that the DEL allele exerts an effect on the stability and levels of HLA-G mRNA (25, 48) and on the levels of soluble HLA-G protein (26, 49), and reinforce the notion that the log-additive model is the one that best fits our study, since the high frequency of DEL/DEL patients (50.9%) implies a strong genetic predisposition of this genotype in the development of gastric cancer, tightly related to a high expression of HLA-G: the more DEL is present in the transcript the more the HLA-G mRNA is stabilized and translated.

Moreover, similar findings have been reported by other authors. Eskandari-Nasab E et al. (47) reported a higher frequency of the 14bp DEL allele and of the DEL/DEL genotype in breast cancer patients compared to the control group. Likewise, Jiang Y et al. (45) reported in a meta-analysis that the HLA-G 14bp DEL allele and the DEL/DEL genotype were associated with increased cancer risk.

#### +3142 C/G Polymorphism

As for the polymorphism +3142 C/G, no statistically significant differences were detected between patients and control subjects although there is a slight increase in the frequency of the C allele in patients compared to controls (**Table 4A**), and an increase in the frequency of the C/C genotype (**Table 4B**); again, this result fits in the proposed hypothesis, since bearing the C allele favors a higher expression of HLA-G (9–11, 28, 30).

In this case, the best inheritance model, based on AIC and BIC values, is the recessive one (**Table 4C**). According to this model, C/C individuals present 2.68 times increased risk of developing gastric cancer. Previous works described that +3142C leads to higher expression of HLA-G (28, 30), due to a lower affinity of different miRNA (148a, 148b and 152) for the HLA-G mRNA. In a similar way to 14bp DEL, which it is described to increase mRNA stability, +3142C/C genotype is overrepresented in patients (30.8% *vs* 14.3%, p=0.017), possibly involving this polymorphism with the development of gastric cancer.

Again, results lending support to our data have been published elsewhere. Jiang Y et al. (50) described in a meta-analysis that the HLA-G +3142 C>G mutation significantly decreased cancer risk, both in the allelic and recessive comparison models.

### Haplotype Formed by the 14bp INS/DEL and +3142 C/G Polymorphisms

Disease susceptibility has long been linked to extended haplotypes of the HLA system (51, 52). The combination of the alleles of these two polymorphisms renders different haplotypes. Assuming the role the variants may have in HLA-G levels and cancer risk, the combination of the DEL and C alleles would pose the highest cancer risk. In fact, and according to the calculations carried out by the SNPStats software, the INS/G combination (considered as the reference value by the software) would be underrepresented in patients, whereas the DEL/C haplotype is significantly more frequent in patients (54.1%) than in healthy controls (44.4%) (**Table 5**). This indicates that the former haplotype is a protective factor, while the latter is a risk factor for this disease. The increase in the frequency of DEL/C haplotype matches the results obtained in the analysis of the individual polymorphisms, suggesting that both 14bp DEL and +3142C variants (that presumably lead to a higher HLA-G expression)are associated with susceptibility to gastric adenocarcinoma. This is, to the best of our knowledge, the first time that an association between the HLA-G 3’UTR region and the development of gastric cancer is disclosed in our population.

### Somatic Mutations

Random mutations take place in cancer, conferring cells a proliferative and invasive capacity and allowing them to escape immune surveillance. In our case, an increase in variants favoring HLA-G expression and thus, abrogating immune response (i.e.: 14bp DEL and +3142C) could be expected in tumoral (T) but not in non-tumoral (NT) distal cells. However, after analysis of paired (T+NT) tissue samples from the 107 patients studied, no somatic mutations were found. The polymorphism present in a T sample analyzed matched that of the paired NT sample in every single patient tested. We can then confidently conclude that the polymorphisms here studied, and their influence on gastric cancer is a pre-existing condition in these patients.

### Survival

Further to mediating disease risk, we measured whether these variants were involved in life expectancy. None of the comorbidities studied were linked to patient survival, whereas TNM staging and 14bp polymorphism revealed a clean association. Disease-specific survival rate (**Figures 3** and **4**) is significantly diminished, and hazard ratio increased (by up to 14-fold) in patients expressing higher levels of HLA-G according to published works (25, 26, 48, 49) (in our case, bearers of the DEL/DEL genotype). Therefore, tumor cells evade the immune system and proliferate unchecked, leading to disease dissemination and death. The 14bp DEL variant has been already associated with worse survival in a cohort of patients with colorectal cancer (39), a tumor with similar histological features to gastric adenocarcinoma, which supports the relevance of this polymorphism in the progression of cancer.

This finding suggests the possibility of considering this molecule as a potential target for therapeutic approaches. Downregulating HLA-G expression with miRNAs (as has been done in other clinical settings) (53–55) or blocking (with monoclonal antibodies) its interaction with cognate receptors (in a way similar to PD-1/PD-L1 current immunotherapies) (56–58), will make tumors visible to immunocompetent cells, eliciting an active immune response.

Although the functional effect of these polymorphisms and HLA-G expression could explain their linkage to disease susceptibility and progression, we cannot, nevertheless, exclude the possibility that these polymorphisms be in LD with other genes (i.e., other class I HLA genes) that could truly mediate the development and prognostic of this disease.

A limitation for this study is that our research is focused on genetic polymorphisms and, although we have confirmed HLA-G expression in tissue and sHLA-G in plasma in part of our cohort of patients (**Supplementary Figure 1**), we could not evaluate the association of HLA-G expression with the risk of developing gastric cancer or the survival of patients.

Further studies, focusing on HLA-G expression on the patients, are required to precisely assess the role of HLA-G in gastric cancer.

Besides these limitations, a more extensive cohort of patients and including other 3’UTR polymorphism (such as +3003 T/C or +3184 A/G) in a larger study would increase the reliability of the associations herein proposed. To ease comparisons between groups, future studies with larger cohorts will include ancestry informative markers (AIMs).

We conclude that the polymorphisms 14bp INS/DEL and +3142 C/G of the *HLA-G* gene mediate gastric cancer risk and survival, and suggest the possibility of establishing new therapeutic approaches aiming at counterbalancing the negative role of this protein in tumors.

## Data Availability Statement

The nucleotide sequences have been deposited to Genbank - accession MZ130952-MZ130955.

## Ethics Statement

The studies involving human participants were reviewed and approved by Comité ético de investigación clínica, Hospital Clínico San Carlos, Madrid, Spain. The patients/participants provided their written informed consent to participate in this study.

## Author Contributions

CV-Y: manuscript writing, investigation, and analysis. IJ: design, analysis, and supervision. MM-A: investigation support and validation. EM-L: analysis. AL-N: investigation support. FS-T: manuscript revision. AG-C, AL-G, IL, and RG: patient follow-up, sample and data collection. AA-V: critical review, project administration, and funding acquisition. JM-V: manuscript writing and revision, supervision, project administration, and funding acquisition. All authors contributed to the article and approved the submitted version.

## Funding

This work was supported by grants from Instituto de Salud Carlos III (PI18/00626 and PI18/00721), with funds from the European Union (Fondo Europeo de Desarrollo Regional FEDER). IJ is a grant recipient of a Universidad Complutense de Madrid—Real Colegio Complutense Harvard grant, (Ayudas para contratos predoctorales de personal investigador en formación CT18/16).

## Conflict of Interest

The authors declare that the research was conducted in the absence of any commercial or financial relationships that could be construed as a potential conflict of interest.

## Publisher’s Note

All claims expressed in this article are solely those of the authors and do not necessarily represent those of their affiliated organizations, or those of the publisher, the editors and the reviewers. Any product that may be evaluated in this article, or claim that may be made by its manufacturer, is not guaranteed or endorsed by the publisher.

## Supplementary Material

Supplementary Figure 1 Membrane bound and soluble HLA-G expression in patients with gastric cancer. **(A)** Representative stainings of HLA-G in tumoral (Tumor 1-4) and distal (non-tumoral) tissue of the patients included in the study. **(B)** Representative measure of sHLA-G expression by ELISA in the patients included in the study (1-37) and supernatants of two HLA-G negative (-) and positive (+) cell lines.
