# Supplementary figures and images for: HLA-G 3’UTR Polymorphisms Are Linked to Susceptibility and Survival in Spanish Gastric Adenocarcinoma Patients
Source: Front Immunol. 2021 Sep 7;12:698438. doi: 10.3389/fimmu.2021.698438 (PMC8453083; doi:10.3389/fimmu.2021.698438)

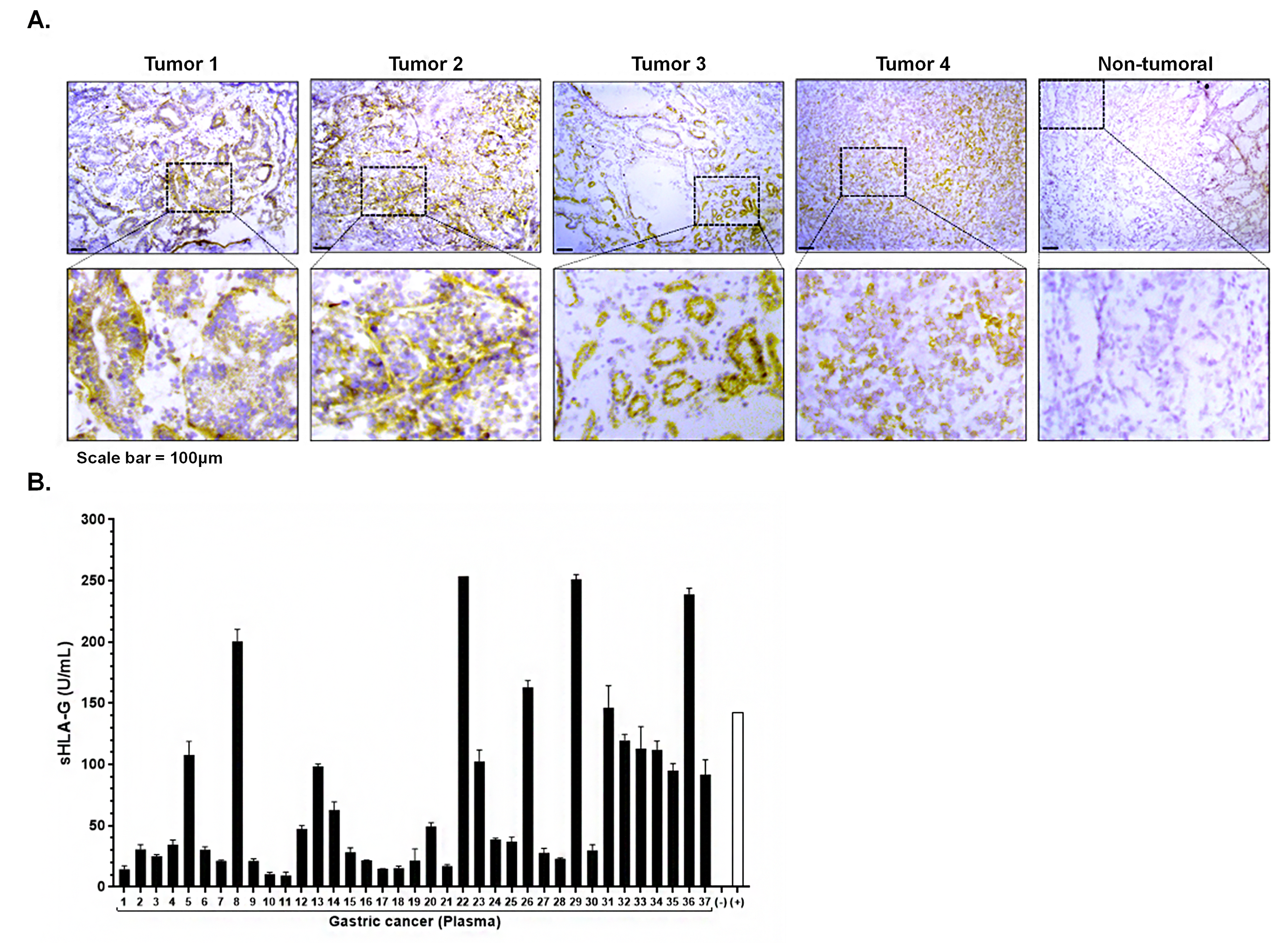

Supplement: Supplementary Figure 1 — Membrane bound and soluble HLA-G expression in patients with gastric cancer. (A) Representative stainings of HLA-G in tumoral (Tumor 1-4) and distal (non-tumoral) tissue of the patients included in the study. (B) Representative measure of sHLA-G expression by ELISA in the patients included in the study (1-37) and supernatants of two HLA-G negative (-) and positive (+) cell lines. [file Image_1.png]
